# Peptide microarray mapping of B cell epitopes on bovine leukemia virus and peptide ELISA analysis of conservation of epitopes in BLV infected Japanese cattle

**DOI:** 10.1186/1742-4690-12-S1-P97

**Published:** 2015-08-28

**Authors:** Lanlan Bai, Shin-nosuke Takeshima, Emiko Isogai, Yoko Aida

**Affiliations:** 1 Viral Infectious Diseases Unit, RIKEN, Wako, Saitama, Japan; 2 Laboratory of Animal Microbiology, Department of Microbial Biotechnology, Graduate School of Agricultural Science, Tohoku University, Sendai, Miyagi, Japan

## 

The bovine leukemia virus (BLV), a retrovirus structurally and functionally related to the human T-lymphotropic viruses HTLV-1 and HTLV-2, is the etiological agent of bovine leucosis. B cell immune mechanisms may play a major role in protection against BLV infection. A battery of 157 synthetic peptides, 15-mer length, 4 amino acids overlapped, was used to mapping B cell epitopes on BLV envelope glycoprotein gp5l and capsid protein by peptide microarrays. Two susceptible cattle with the homozygote BoLA-DRB3 *1601 allele and two non-susceptible cattle with DRB3 *1501/*2703 alleles and DRB3 *1501/*0503 alleles were infected with BLV, and serums were collected from those cattle each of having challenged BLV before and after. Only one epitope A out of 157 synthetic peptides responds with all of four BLV positive serums not their negative serums. To demonstrate whether epitope A is common B cell epitope or not by our established peptide ELISA system that uses maleimide activated mariculture keyhole limpet hemocyanin (mcKLH) carrier protein. To found, there are 7 kinds of BLV positive serum no response with epitope A among 232 kinds of positive serum. Furthermore, we searched other peptides respond with BLV positive serum that contain 7 kinds of serum no responded with epitope A and found epitope B among peptides of non-specifically responded on peptide microarray. Epitope B strongly responded with all of 232 kinds of serum as estimated by peptide ELISA system. Our results shows that epitope B has a tendency to react some BLV negative serums, thus we will perform further experiments to determine the common B cell epitope just responses with positive serum by peptides that are 3 alanine substitutions as shifting one amino acid on epitope sequence.

